# Supplementary material for: Indoor noise level measurements and subjective comfort: Feasibility of smartphone-based participatory experiments
Source: PLoS One. 2022 Jan 27;17(1):e0262835. doi: 10.1371/journal.pone.0262835 (PMC8794191; doi:10.1371/journal.pone.0262835)
Supplement: S1 File — (DOCX) [file pone.0262835.s001.docx]

Supplementary Materials for

Indoor noise level measurements and subjective comfort: feasibility of smartphone-based participatory experiments

Carlo Andrea Rozzi, Francesco Frigerio, Luca Balletti, Silvia Mattoni, Daniele Grasso, Jacopo Fogola

Correspondence to: carloandrea.rozzi@nano.cnr.it

**This file includes:**

Supplementary Text

S1-S11 Figs

S1-S2 Tables

Supplementary References

# Supplementary text

## Measurement protocol **(translated from the original in Italian)**

**Phase I**: preparation.

1. Install the OpeNoise app.
2. Skip the calibration;
3. become aware of the acoustic environment of the participant’s home and acquaint yourself with the app by performing casual measurements at day and night-time;
4. identify locations and times at which most often the maximum and minimum acoustic comfort is experienced.

**Phase II**: “measuring noise”

1. For each situation (i.e. time and location) identified during Phase I perform an indoor sound level measurement according to the protocol described below.

**Phase III** (optional): “measuring silence”.

1. Identify the home’s most quiet location and time (typically a bedroom at night with windows shut);
2. perform a 1 min measurement of the background noise.

**Measurement protocol** for each chosen location/time:

1. Position yourself indoor at 1 m from an open window in the chosen room;
2. disable automatic screen switch-off and enable do-not-disturb mode;
3. silence indoor noise sources (TV, animals, voices, music, etc.);
4. identify the smartphone mic and point it towards the window (enable in app reverse screen mode, if convenient);
5. repeat the following sequence:
   1. stay quiet throughout the measurement;
   2. press reset and record for at least 1 minute (max 2 minutes);
   3. read and record the values of LAmin, LAeq(t) and LAmax;
   4. in case of undesired noise repeat from 5a;
6. annotate all the fields in the provided table including measured values, information about the location and time, a subjective rating of sound comfort during the measurement in a scale from 1 (less comfortable) to 5 (more comfortable) and comments;
7. when a sufficient number of observations were collected, select the two corresponding to the most comfortable and least comfortable states (hereby simply named “noisy” and “quiet” states) experienced and send the data via the provided google form.

## Citizen science initiatives at Cnr

The initiative is part of a larger project called “Scienza sul balcone” (science on the balcony) [1] born in Italy in the spring of 2020 from an idea by Alessandro Farini (National Institute of Optics of the National Council of Research of Italy) and Luca Perri (astrophysicist and science communicator), during the national COVID19 lockdown - which lasted from 11 March to 3 May 2020. In that period, flash mobs - born spontaneously and quickly spread throughout Italy through social networks and instant messaging services - were organized several times, in which citizens gathered at their windows or on their balconies to realize together with simple actions. One of these events required pointing the flash light of the smartphone towards the sky in the evening with the aim of illuminating the night sky and being photographed by one satellite. In the following days images of extraordinarily illuminated Italy photographed by satellite were also shared on social networks, but in reality the brightness of the flash light of a smartphone cannot be detected by a satellite, and the images were normal nighttime satellite images retouched to make the Italian peninsula unusually illuminated. This unusual flash mob, however, gave an idea to Farini and Perri, who decided to propose a new flash mob, a scientific experiment concerned with measuring the intrusive light that enters homes at night using the smartphone's brightness sensor but also to sensitize the population to pay attention to the scientific truthfulness of the news disseminated through social networks. This first experiment had great media coverage, both nationally and internationally [2]. The present initiative is the second experiment in the series, and more are being organized on different topics.

## Communication campaign

Thanks to the involvement of the Cnr Communication and Public Relations Unit - which for the first time collaborated with the scientific network to carry out a citizen science project of this magnitude on the national territory - a targeted communication campaign was prepared, summarized briefly by points below:

- **naming**: a representative and easily identifiable name was chosen through a hashtag, which immediately went viral on social networks and in general news;

- **catchphrase**: to highlight the shared component of the experiment, an engaging and participatory slogan was chosen: "Have you ever participated in a scientific experiment directly from your home?”;

- **graphics**: a simple and minimal logo was chosen, simple images suitable for sharing via social networks;

- **web page**: it was decided to create a simple web page in which the data collection system of the experiment was inserted without privacy problems and containing instructions, a series of insights and a map of the measurements entered;

- **identification of communication tools** suitable for the purpose: social channels (FB, YT, IG), promotion on the institutional website of the Cnr institutes involved and the communication portal, involvement of the media through press releases, collaboration with important Italian scientific festivals.

As a result, there have been numerous releases in the media (TV, radio, print media and online newspapers). During the year, citizen science was mentioned several times in the newspapers and there was talk of #scienzasulbalcone. Interactions in the official and social pages of the Communication and Public Relations Unit of the Cnr increased during the periods in which experiments were carried out. The live broadcasts made to present the experiments were among the most visible videos of the year in the YouTube channel and on the Facebook page of the Unit.


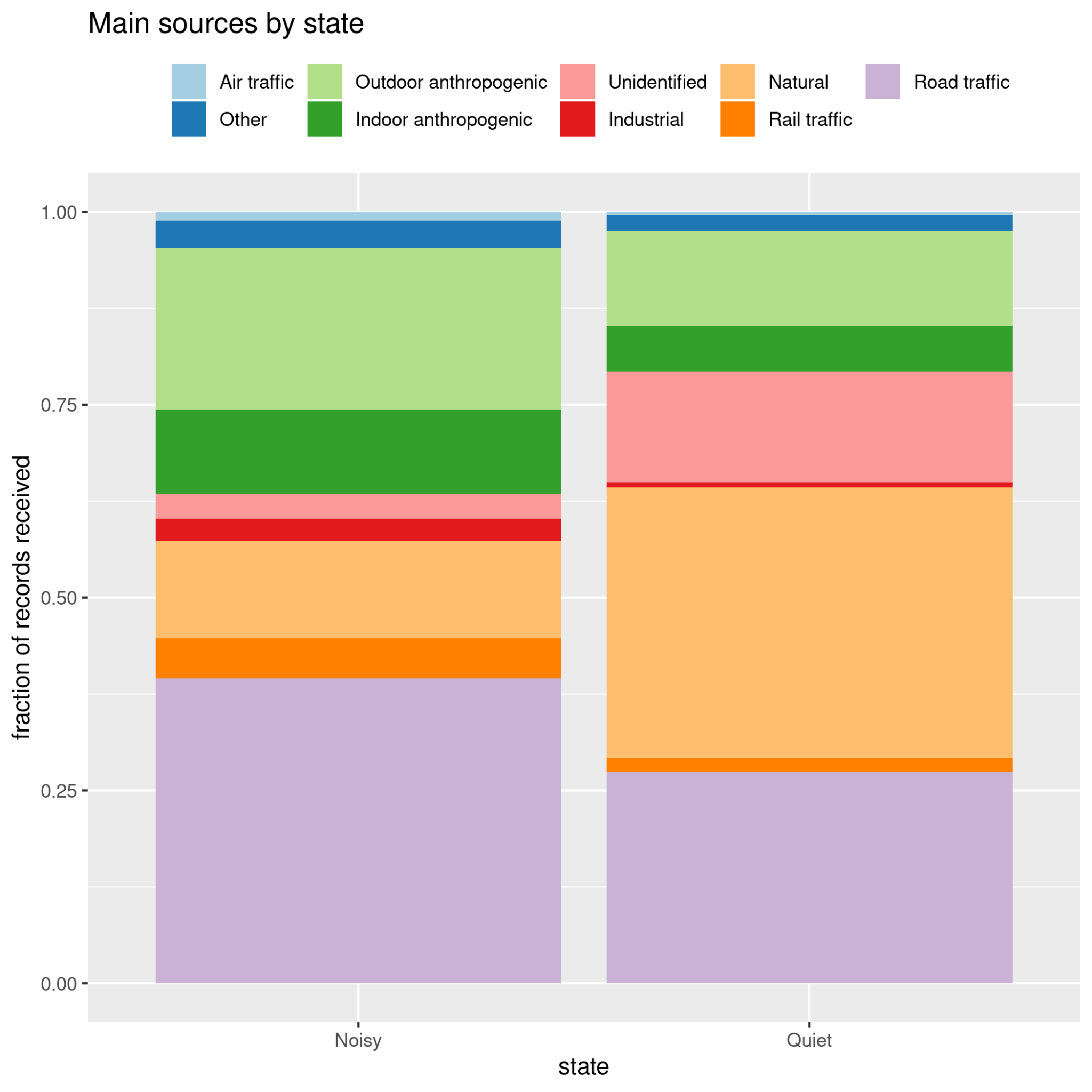


**S1 Fig. Main sources of noise in the low and high comfort states.** In the high comfort state less traffic, industrial and anthropogenic sources are reported, while the number of observations related to natural and unidentified sources increases.


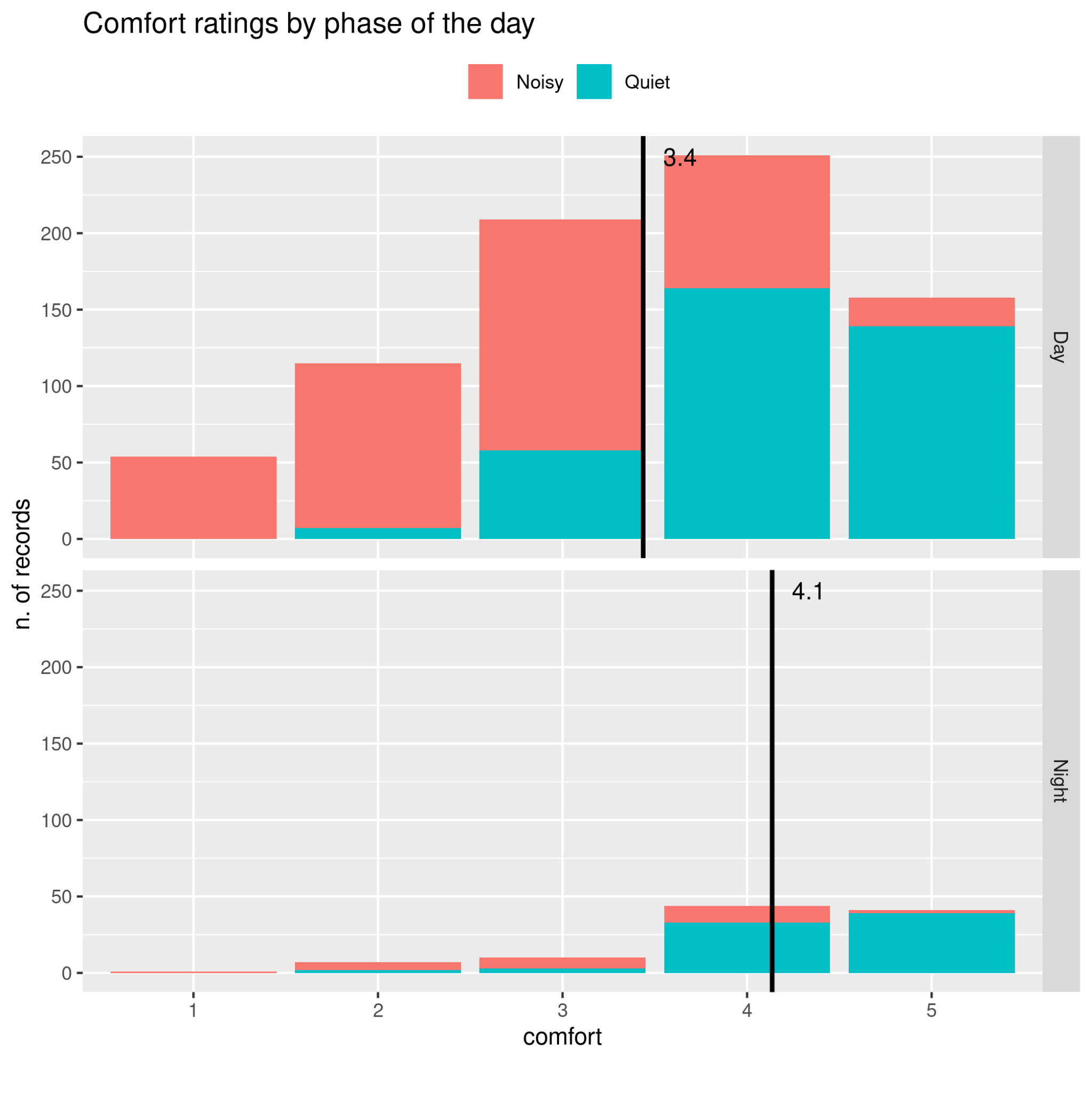


**S2 Fig. Comfort ratings during night (10pm-6am) and day time (6am-10pm).** The colors indicate the number of records reporting a high comfort or low comfort state. Black lines and labels show the mean value of the scores. The mean comfort during day-time (*N = 787, M* = 3.4, *SD* = 1.2) is lower than the mean comfort at night (*N* = 103, *M* = 4.1, *SD* = 0.9).


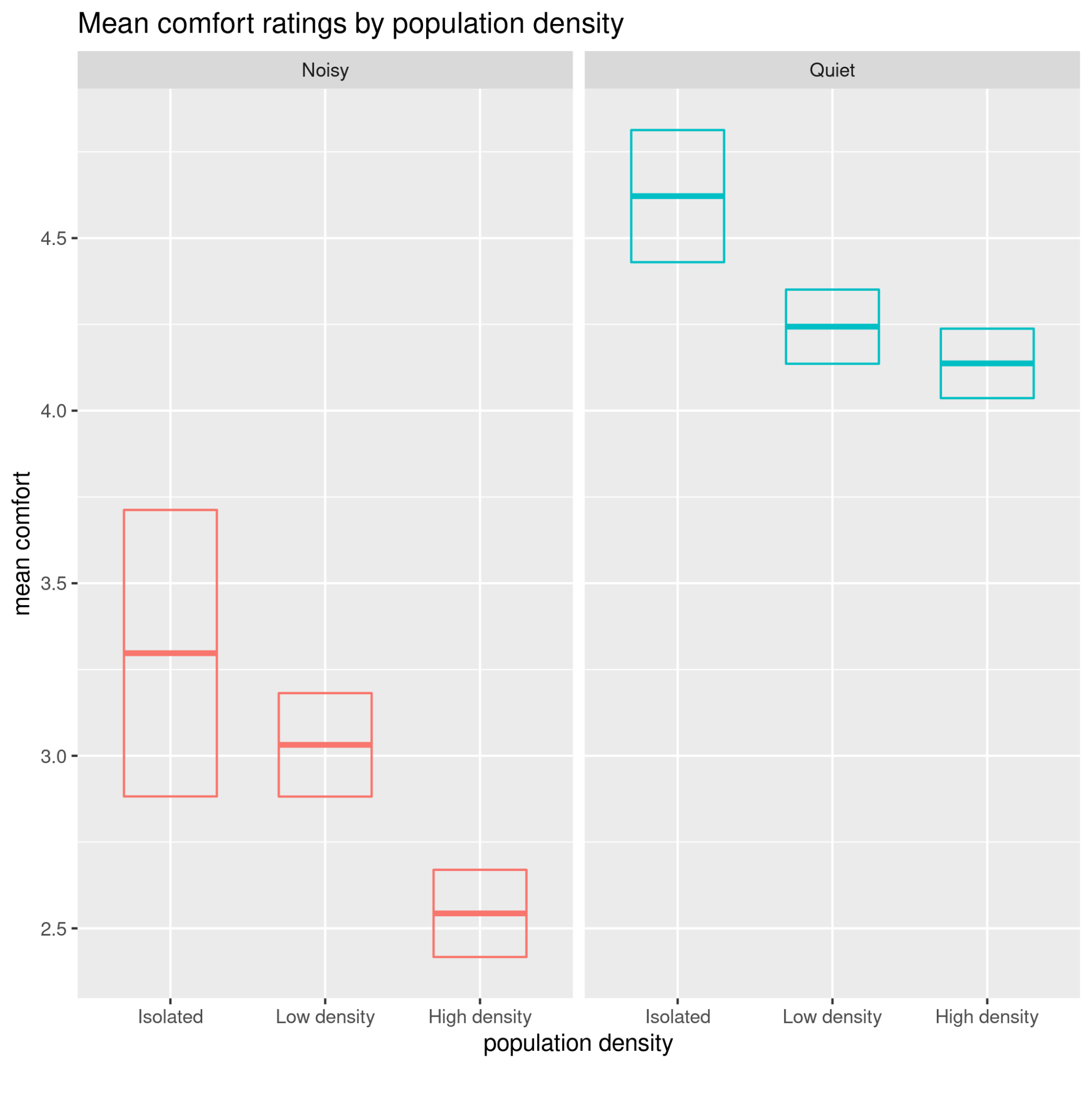


**S3 Fig. Mean values (thick lines) and 95% confidence interval (boxes) of comfort ratings by population density and comfort state.** A linear mixed effect model with state and population density as fixed effect and participants as random effect shows that comfort depends both on the state and the population density. The isolated places mean comfort rating is lowered by 0.32 in low density places and by 0.62 in high density places (both with standard error 0.12). The mean difference between quiet and noisy comfort is 1.41 ± 0.06 (standard error). Mann-Whitney U tests show that the distributions of isolated and highly populated places always significantly differ (*p* < 0.001 quiet, *p* = 0.002 noisy). The mean comfort of low-density places instead does not differ significantly from the one of high-density ones in noisy states (*p* = 0.3). It does not differ significantly from the one of isolated places in quiet states (*p* = 0.1).


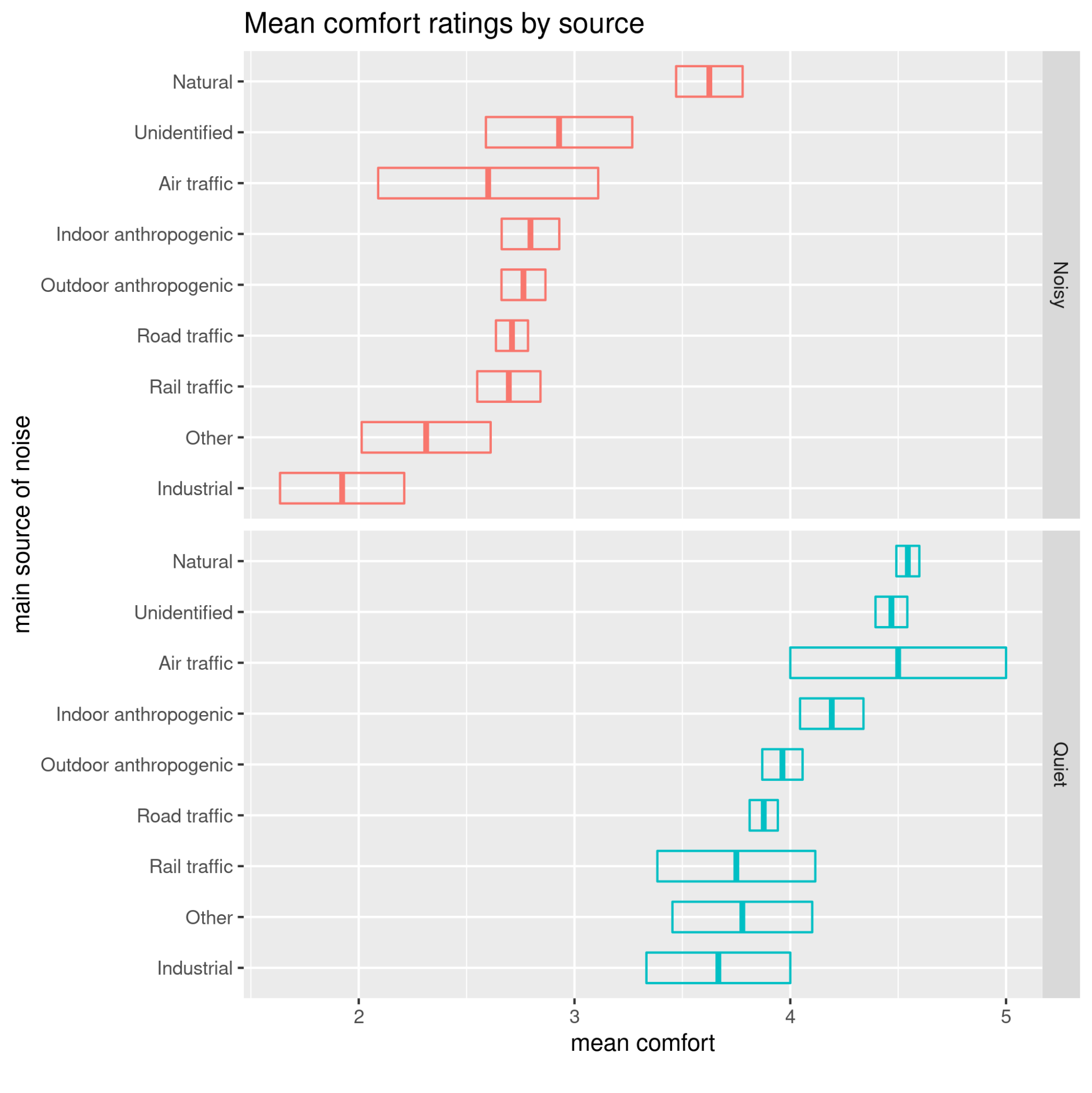


**S4 Fig. Mean comfort ratings (thick lines) and standard errors (boxes) by source and comfort state.** The lowest mean comfort score is always attributed to industrial sources (*N* = 13, *M* = 1.9 for noisy states and *N* = 3, *M* = 3.7 for the quiet states). The highest is always attributed to natural sources (*N* = 60, *M* = 3.7 for noisy states and *N* = 160, *M* = 4.5 for quiet states).


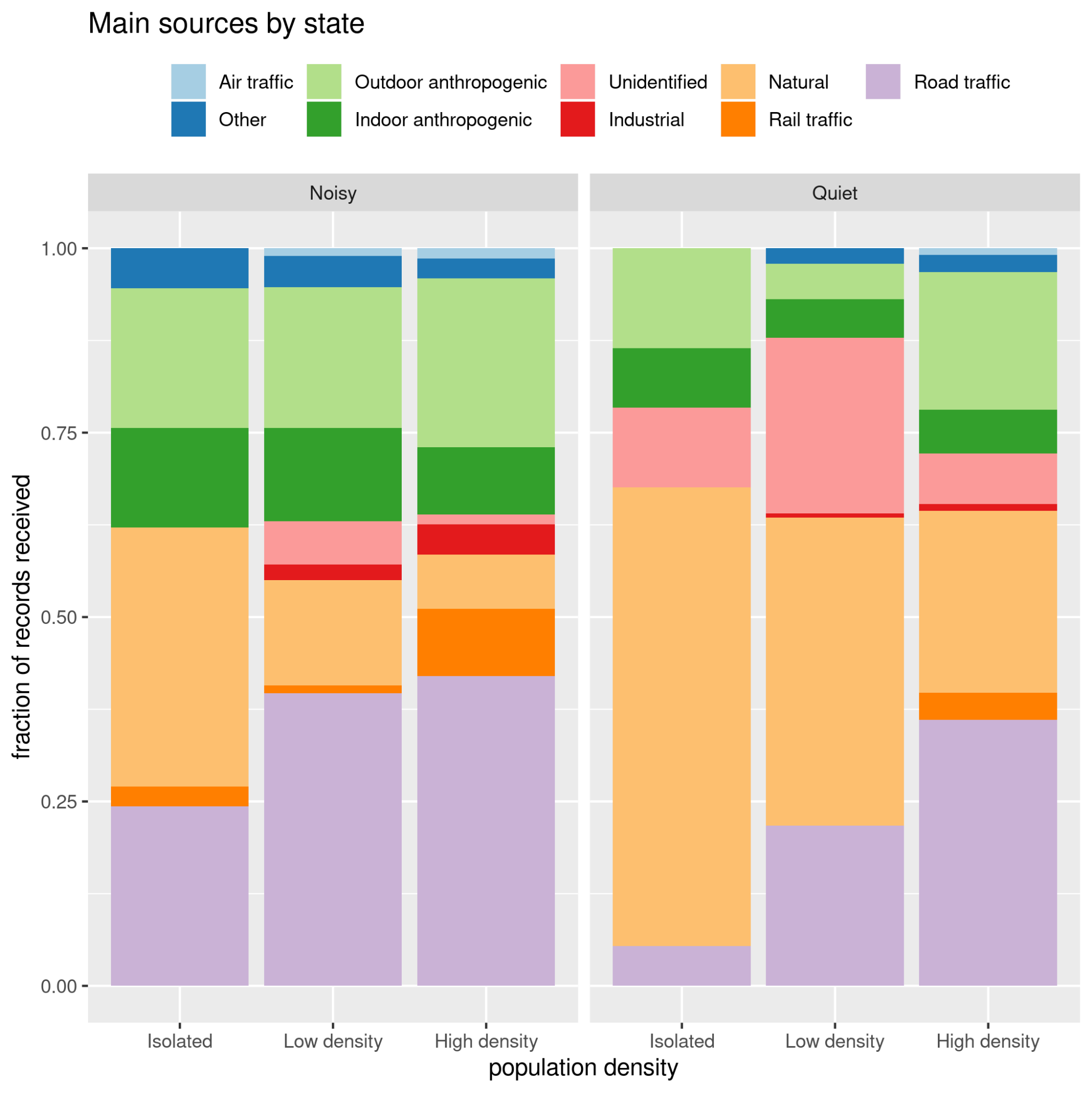


**S5 Fig**. **Differences in the frequencies of main sources of noise among places with different population density.** The frequencies of road traffic related noise increases monotonically with the population density in the neighborhood of the measurement place both in noisy and quiet situations. Natural sources show the opposite trend. Indoor anthropogenic sources are cited with almost constant frequencies in all environments and situations. Noise of undefined source is more frequently reported from low-density and Isolated places in quiet situations.


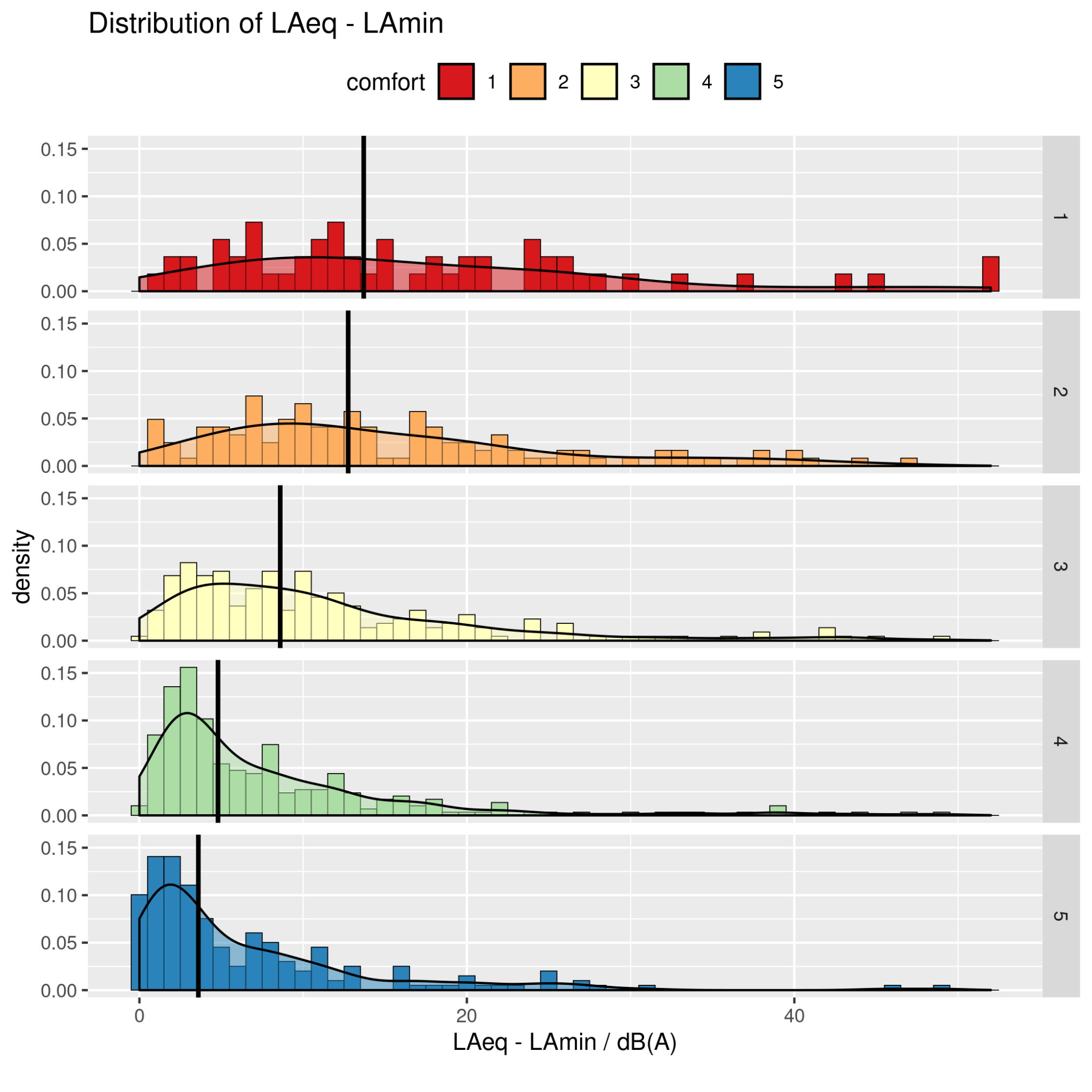


**S6 Fig.** **Distribution densities of *LAeq - LAmin* at different comfort levels.** Black lines indicate median values of the distributions. Both median and interquartile range decrease with increasing comfort from a maximum of 13.7 and 16.5 *dB(A)* respectively to a minimum of 3.6 and 7.0 *dB(A)* respectively.


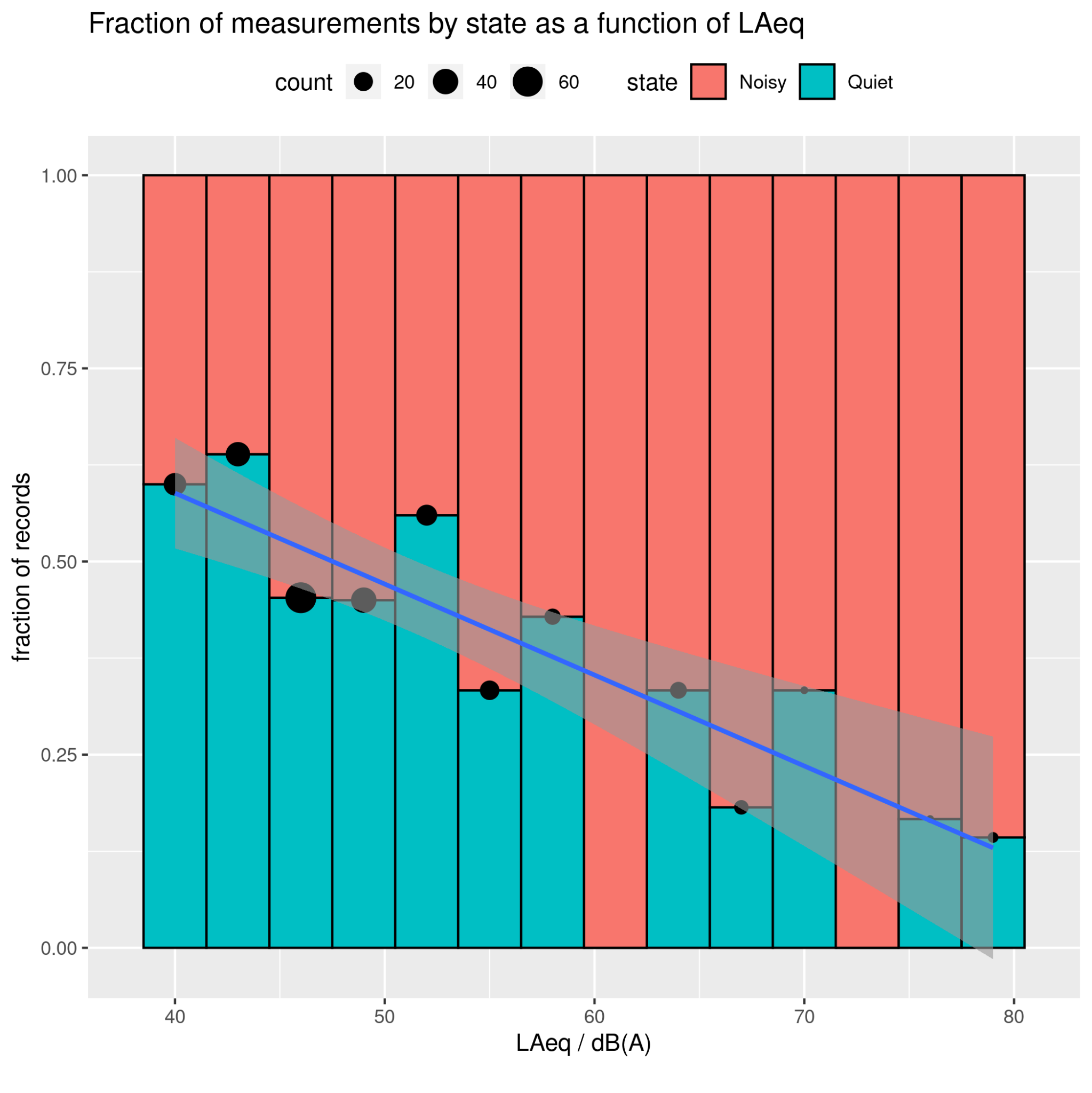
**S7 Fig**. **Fraction of measurements referring to noisy or quiet states as a function of LAeq in 3 *dB(A)* wide bins.** The dot size indicates the number of observations per bin. The blue line indicates the linear relationship$P\left[ \text{not quiet} \right]\left( \text{\%} \right)=1.08LAeq$obtained by a linear model (*F*_(1,11)_ = 738, *p* < 0.001, R^2^ = 0.985) with the number of observations per bin as weights. The shaded area indicates the 95% confidence interval of the linear estimator.Below LAeq = 46 *dB(A)* more measurements are referred to quiet states than to noisy states; above this threshold more measurements are referred to noisy states.


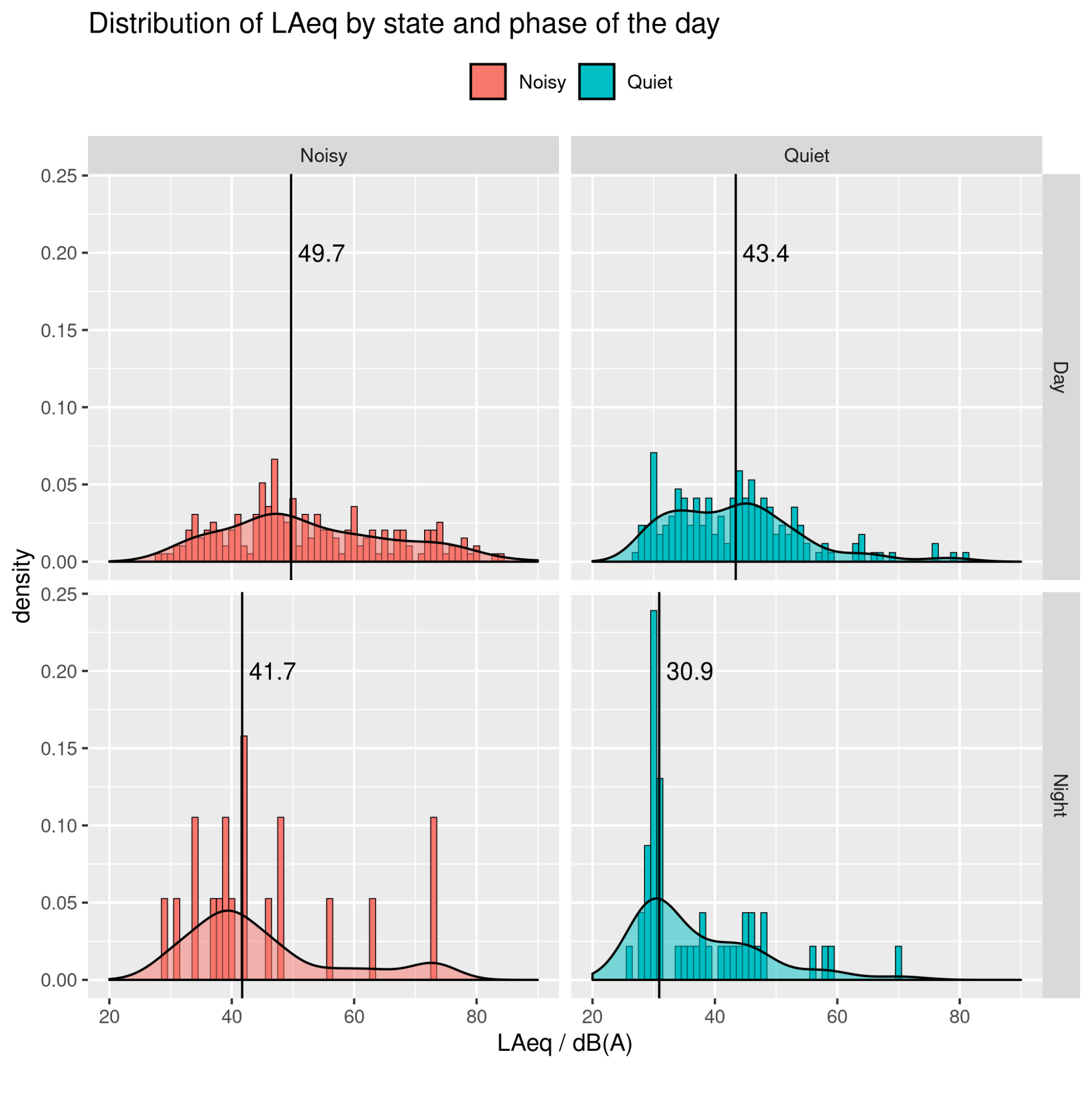


**S8 Fig. Distributions of LAeq by night/day phases and noisy/quiet states.** Vertical black lines and the numeric labels indicate LAeq median values.


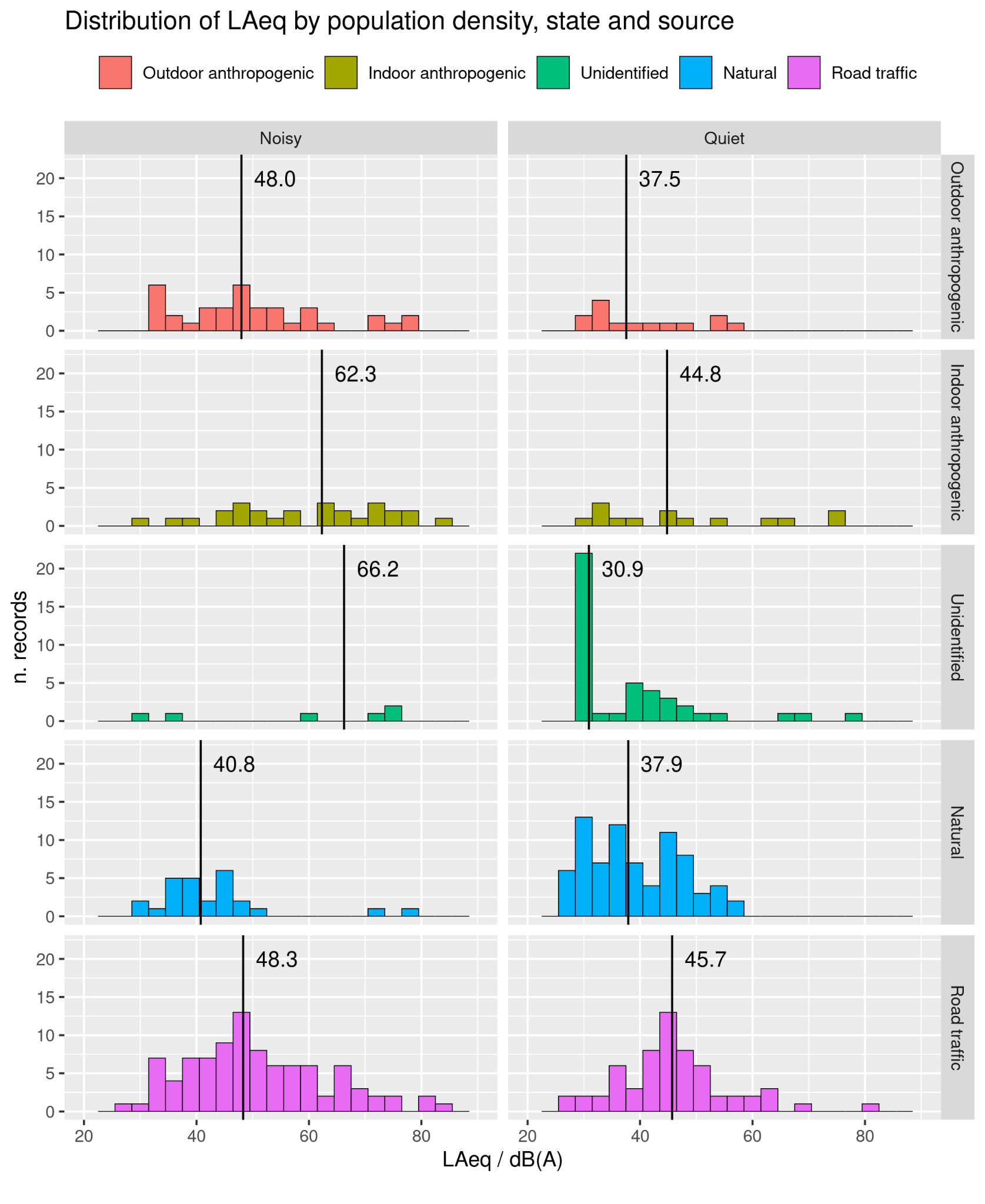


**S9 Fig.** **Distribution of LAeq by states (columns) and source (rows).** Only the sources with the largest number of reports are included. Black lines and numeric labels point at median values.
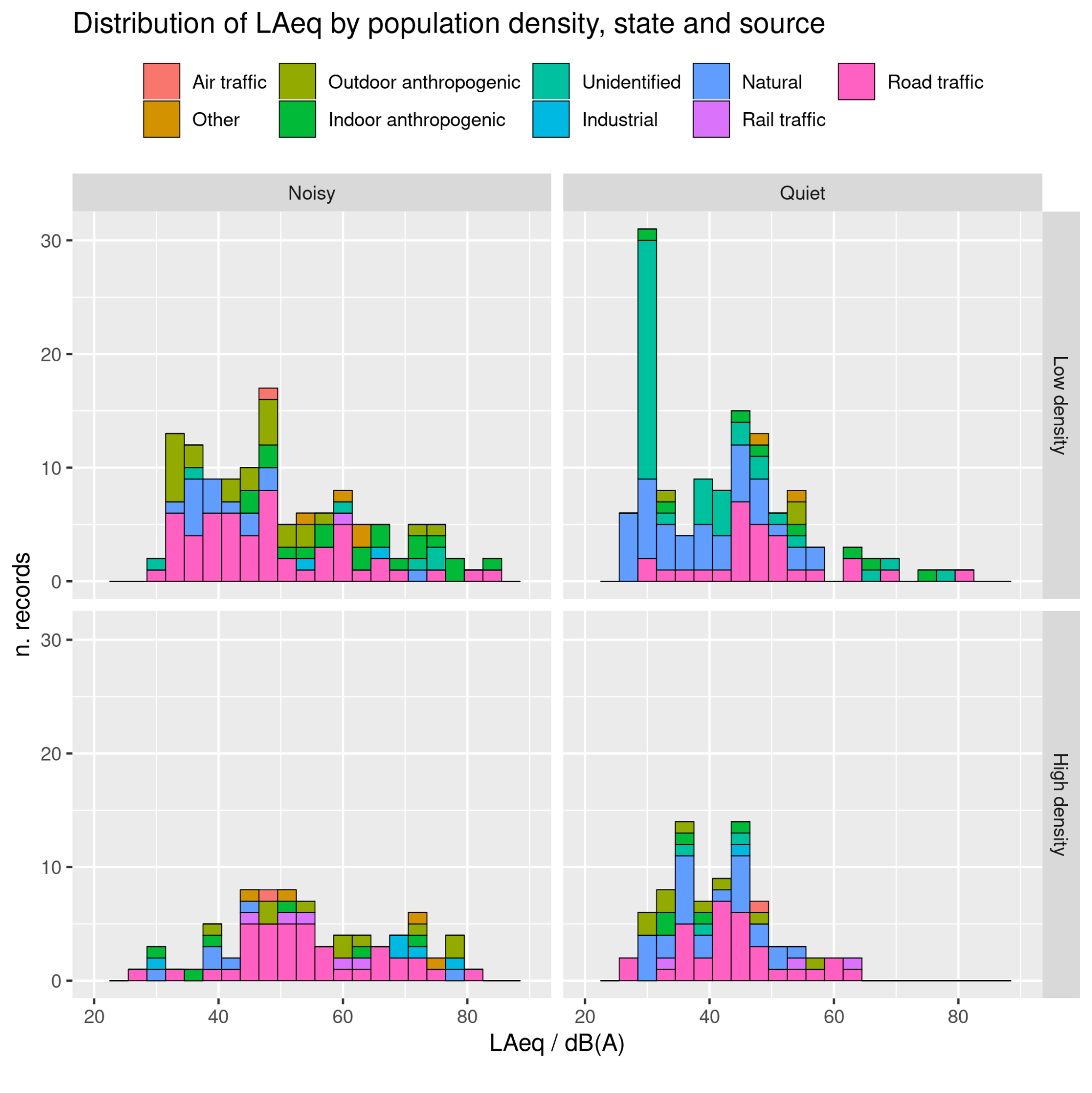
**S10 Fig.** **Distribution of LAeq by states (columns) and population density (rows).** Isolated places are not shown since their number is negligible. The colors indicate the sources of noise in each 3 *dB(A)* wide bin.


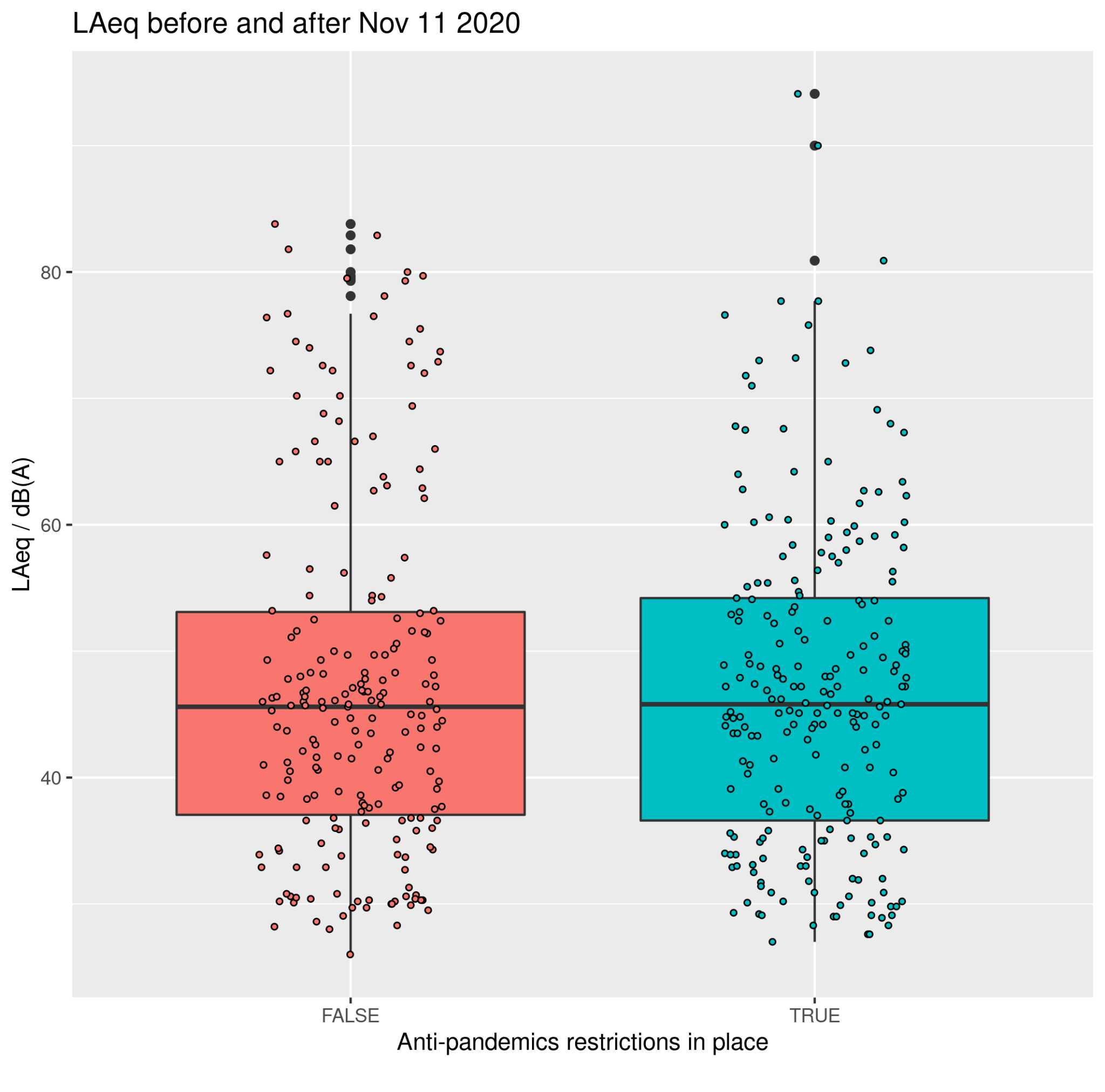
**S11 Fig. Average noise levels before and after Nov 11.** On Nov 11 2020 a set of limitations, mostly to road-traffic and activities during night, was introduced in some Italian regions. The observations in our experiment were mostly performed during day time and do not show significant variations in LAeq. Before Nov 11: *N* = *211*, *Md* = *45.6 dB(A)*; after Nov 11: *N* = *225*, *Md* = *45.8 dB(A)*. Independent samples t-test *t_(424)_* = *0.39*, *p* = *0.6*.

|  | **mean comfort** | | **mean LAeq (*dB(A)*)** | |
| --- | --- | --- | --- | --- |
| **sample size** | **Noisy** | **Quiet** | **Noisy** | **Quiet** |
| 890 | 2.81 | 4.22 | 58.4 | 42.5 |
| 760 | 2.83 | 4.23 | 58.3 | 42.4 |

**S1 Table. Mean values of comfort and LAeq in two samples with different sizes.** The smaller sample was obtained by removing 65 values that were mistakenly uploaded in the wrong order in the online form.

| **Lab calibration using an external microphone** | | | | |
| --- | --- | --- | --- | --- |
| **producer** | **model** | **OS** | **version** | **gain / *dB(A*)** |
| Apple | iPhone 6 | IoS | 12.01.03 | 24.0 |
| Apple | iPhone 7 | IoS | 12.01.02 | 23.6 |
| Apple | iPhone 7 plus | IoS | 12.1.2003 | 24.0 |
| Apple | iPhone SE 1 | IoS | NA | 24.0 |
| Apple | iPhone XS | IoS | 12.01.03 | 24.0 |
| Honor | Honor 9 | Android | 8 | 9.5 |
| Huawei | Ascend G7 | Android | 4.04.04 | -2.5 |
| Huawei | Ascend P8 | Android | 6 | 17.0 |
| Samsung | Galaxy A20 | Android | NA | 21.0 |
| Samsung | Galaxy A5 | Android | 7 | 11.7 |
| Samsung | Galaxy A8 | Android | 8 | 13.6 |
| Samsung | Galaxy J7 | Android | 8.01.00 | 10.0 |
| Samsung | Galaxy S10 | Android | 9 | 15.8 |
| Samsung | Galaxy S7 | Android | 8 | 15.0 |
| Samsung | Galaxy S8 | Android | 9 | 14.6 |
| Samsung | Galaxy S9 | Android | 9 | 16.0 |
| Xiaomi | Redmi Note 8 | Android | 9 | 2.5 |

**S2 Table.** **Smartphone calibrations used in this work.** The gain is the value to be added to the raw LA values to obtain absolute noise levels.

# Supplementary References

[[1] Consiglio Nazionale delle Ricerche, "#scienzasulbalcone” [Internet].](https://www.nature.com/articles/d41586-020-00154-w) <https://sites.google.com/view/scienzasulbalcone/> (2020).

[2] Nature News, "Thousands of Italians take part in citizen-science project". *Nature* 31 March (2020). Available from <https://www.nature.com/articles/d41586-020-00154-w>
