# Supplementary material for: Indoor noise level measurements and subjective comfort: Feasibility of smartphone-based participatory experiments
Source: PLoS One. 2022 Jan 27;17(1):e0262835. doi: 10.1371/journal.pone.0262835 (PMC8794191; doi:10.1371/journal.pone.0262835)
Supplement: Attachment — Submitted filename: response_to_reviewers.docx [file pone.0262835.s002.docx]

Response to reviewers

We thank the reviewer for making detailed and constructive comments.

We have substantially edited our manuscript to take into account all of the suggested issues. In the revised manuscript, relocated text or minor edits are highlighted in green; newly added or heavily modified text is highlighted in yellow.

In detail, we moved the differential analysis from the Supplementary Information to the main text. We expanded the methods section and better stressed the aims, scope and limitations of our work in the title, abstract, introduction and conclusions. We have more clearly distinguished results from discussion. We made linguistic corrections throughout and formal changes to comply with PlosOne format requirements.

Moreover, we have performed additional data analysis and, for full transparency, we made the experiment data publicly available via the Open Science Foundation at the URL [https://osf.io/nufmx](https://osf.io/nufmx/) .

Please find below our detailed point-by-point replies to the reviewer’s comments.

## Reviewer’s comment 1

*TITLE: please consider title on the lines of ‘Feasibility of -------------- ‘.*

## Authors’ reply

We have modified the title as suggested by the reviewer. We have further stressed the concept of feasibility in the introduction.

## Reviewer’s comment 2a

*INTRODUCTION: Rationale of the study needs to be strengthened. Many issues cited below needs to be addressed by the authors:*

*a) Although the authors discuss on various apps available for indoor noise measurements, and cite the limitations, it is very general. No specific data on reliability of their claims is available, specific to the apps cited. The authors have missed on the test-retest reliability, or accuracy of the cited apps in noise measurements.*

## Authors’ reply

We have expanded the introduction and commented on the testing we performed on the OpeNoise app. We cited the relevant literature reporting both software and hardware comparisons of other apps that were not used in our experiment.

## Reviewer’s comment 2b

*b) The rationale on geospatial tracking breeching the privacy is not a strong reason, as the participants are asked to sign the informed consent on all the apps cited in introduction, before their usage. The terms and conditions of the spatial tracking would have been brought to their notice under disclaimer.*

## Authors’ reply

We toned down the relevant sentence in the introduction as we meant to express more a concern than a rationale for the whole work. We left mention of this issue, though, because, while it's true that asking consensus to geotrack participants formally settles the problem from a merely legal point of view, yet, most people (at least in the EU) prefer not being tracked by apps at all, especially if tracking leads to determining their home address. Therefore we believe that geotracking should be avoided as much as possible when addressing the largest possible audience as this choice might substantially affect the penetration of citizen science initiatives similar to this one.

## Reviewer’s comment 2c

*c) The DATA was collected in 2020, during the nation-wide lockdown of Italy. Hence, the noise levels in the inside house will be more (as more worked from home) and surrounding neighborhood activity would have considerably increased (say houses in the flats like residencies). The authors themselves point out this limitation in Introduction section (Page 9, lines 66-68) that the data was collected in Pandemic times. In my view, the data hence will not be representative of the day-to-day regular activity, which is the cause of concern for publication.*

## Authors’ reply

Anti-pandemic restrictions in Italy underwento several phases. The first, strictest and nation-wide, was imposed between Feb and Apr 2020. In the remainder of the year a regional semaphore scheme provided for several levels of limitations different from region to region.

Please note that our experiment was **not** performed during the general lockdown. Restrictions potentially affecting our data were enforced in some regions starting from Nov 11. They were way less severe then the ones for a complete lockdown. In particular both road traffic and most working activities were **not** limited during day-time (see Prime Minister Decree DPCM Nov 3 2020 available from <https://www.gazzettaufficiale.it/eli/id/2020/11/04/20A06109/sg> ).

Moreover, 89% of all the observations were performed during daytime, and about half of them were performed by our participants **before** Nov 11 (i.e. with virtually no mobility restrictions). Therefore, although it is possible that differences were noticeable locally, it is not obvious that any difference could be seen on the average noise levels.

This situation is actually well reflected in our data: there is no evidence of changes in the average LAeq before and after Nov 11.

We have removed a sentence that might erroneously be interpreted as if people were all confined at home during the time of the experiment. We explained better the restrictions actually in place in the discussion section and we added a picture to the Supplementary information showing that no differences emerge from our data between *pre* and *post* Nov 11 in the average LAeq.

## Reviewer’s comment 2d

*d) The data collected was emailed to the experimenters. The participants were asked to send the representative data of the nosiest and a quietest situation. This would induce bias and lead to perceptual judgement (if participants chose not to send a particular scenario based on the sound quality). Authors have not cited how this issue was controlled.*

## Authors’ reply

Of course we are aware that the participants' choices are not representative of the time-averaged comfort of their homes. In order to measure this aspect a larger number of measurements would be necessary, which is hardly obtainable in an experiment meant for the general public. The request to choose the two observations corresponding to “extreme” situations out of a possibly larger set was mainly dictated by feasibility considerations. Experience shows that the lengthier and more complex the procedures are, the less collaborative participants are.

However, we restate that our aim was not to obtain such a fine-grained mapping, but rather to correlate subjective comfort and noise levels. Under this respect, perceptual judgement is actually a feature of our experimental design, and considering just the two extreme values is indeed informative, as it guarantees a better noise-to-information ratio in a situation in which large variance in the data is expected.

We have better highlighted the scope of our experiment in the introduction to clarify what kind of information **cannot** be inferred from our data.

## Reviewer’s comment 3a

*METHODS:*

*a) The reliability of the smart phones/ android devices in noise measurements is cited in general in introduction chapter. However, the qualitative differences in recording due to differences in handsets configurations across participants is not controlled. Additionally, the microphone sensitivity and its frequency resolution were not controlled across participants, which induces an inherent variability in data.*

## Authors’ reply

In an unsupervised citizen-contributed set of measurements a large amount of variability is always to be expected. Our main contribution here is to show that, **despite** this variability, meaningful information can be extracted by such experiments, provided some aspects are handled with care.

Please note that all the calibration gauges we applied were carefully obtained in a lab environment before performing the experiment after assessment of the corresponding phone model. The results of this preparatory work are a crucial part of our work, as they allow us to point out what were the most reliable handsets in the mix, and to restrict the analysis to improve accuracy.

We have revised the manuscript by addressing potential sources of variability in the data both in the results section and in the conclusions. Moreover we have performed a new analysis of the model-related variance in our data. Please also see the reply to comment 4a about this.

## Reviewer’s comment 3b

*b) How was the distance aspect from the source in the room controlled. Eg. Say, the INVERTER being the highest noise generating object. In this case placing the smart phone at 30 cms from the source to 1 meter from the source will drastically affect the representative sample, although in both cases inverter generated noise may be the highest. Authors have not reported of any mechanisms to control such biases. Placing 1 m from the open window is the only condition mentioned, how abt the noise source. No information given.*

## Authors’ reply

In establishing the measurement protocol we considered the Italian regulation as specified in the Ministerial Decree March 16 1998. It provides a set of rules for (professional) noise level measurements some of which we imported in our protocol. Among them the choice of a distance of 1 *m* from the open window is motivated by the fact that the measurements are meant to estimate disturbing noises **coming from outside**, not from inside the lodging unit. The distance of the source of external noise, for a given value of LAeq, is not a factor affecting comfort.

About internal sources of noise, please consider that, in our protocol, it is clearly stated that any source of disturbing noise from inside the home (e.g. TV, voices, music, animals, etc.) must be muted, or the measurement must be repeated, in case of unforeseen disturbances.

Finally, residual noise (i.e. background noise from indoor sources such as heating) is taken care of by performing differential measurements. By measuring noise level with and without the disturbing source (i.e. what we briefly call the “noisy” and “quiet” situations) all the residual sources of internal noise are removed from consideration, as only differences in noise levels matter. It is also worth mentioning the fact that heating in Italy is mostly provided by water-circulating radiators (powered by a gas-fuelled boiler normally not located in living rooms), while hot-air or heat-pump heating is very rare in private buildings.

In the revised manuscript we have stated the points above in the methods section. Moreover, thanks to the reviewer’s comment, we realized that the importance of differential measurements and their analysis was somewhat obscured in our first version of the manuscript. Therefore we have moved the relevant figure from Supplementary Information to the main text and expanded the discussion therein.

## Reviewer’s comment 3c

*c) Although the ambient noise was measured in few participants, the results of these findings or analyses corresponding to the same is not reported.*

## Authors’ reply

If the reviewer is referring to the measurement of background noise indoors in the quietest possible situation, this corresponds to the optional measurement indicated as Phase III in our protocol. About half of the participants performed this measurement. We **did** report on these findings. Fig. 3 (revised manuscript numbering) shows the outcomes after calibration.

If “ambient noise” is referred to residual background noise (i.e. noise remaining after the sources of disturbance are removed), we realized that this differential analysis was somehow overlooked in the first version of our manuscript, and we added it to the revised manuscript. Please also see the reply to comment 3b.

## Reviewer’s comment 3d

*d) The participants were asked to read and record the values of LAmin, LAeq(t) and LAmax; based on which the subject would have chosen the sample to email. If the subjects were to use perceptual noise judgements to send the samples, choose which is noisiest and quietest, asking them to record the values would induce bias on their comfort ratings.*

## Authors’ reply

Participants were actually **not** asked to send measurements **based** on the values of LA. They were instructed to sample their environment, to identify extreme situations solely according to their subjective judgement of comfort, and **then** to measure LA in each situation with the app. Please check the measurement protocol in the Supplementary Information. Moreover, participants were clearly informed that they would be using uncalibrated smartphones and therefore the LA values they read did not correspond to "true" values, so reverse bias is truly unlikely. Anyway we checked that no obvious correlation exists between calibration value and reported comfort.

Perceptual judgement is actually a driving feature, not an issue, in this work, the aim being to check if it could be correlated with calibrated measurements of LAeq (see also reply to comment 2d).

To make these point more transparent we have modified the abstract and we stressed the aims of this work in the introduction.

## Reviewer’s comment 3e

*e) How did the authors ensure that the participants followed the instructions and the environmental influence on noise recording. Eg. incomplete opening of window, carpeting in the room, sound absorbers in room etc. A considerable variability can stem from them, which is not discussed.*

## Authors’ reply

We took the following measures to minimize data degradation: adherence to the measurement protocol was promoted by sharing clearly written material, and actively calling for the involvement of teachers and schools in the communication campaign. This allowed for a "proximity layer" of informed people (i.e. teachers) to assist students more closely and motivate them. Both aspects were key to ensure that the rules are followed as accurately as possible. Moreover, data redundancy, and careful cross-checking of the data allowed us to discover a number of inconsistent data that were removed from the set.

We are well aware that full compliance to the instructions cannot be 100% guaranteed in **any** unsupervised participatory experiment. The motivation (and challenge) of our work was precisely to check if meaningful data could be obtained with a carefully designed protocol **despite** *a priori* expecting a large interindividual variability (partly due to non-compliance to the rules).

Factors such as room furniture or sound-proofing are not relevant in our research, as the focus was measuring intrusive noise and subjective comfort at any location, not sound isolation qualities of the lodgings. The inner details of participants’ measuring location are cancelled out when performing the analysis of differential noise levels (see also reply to comment 3b).

We have added an estimation of both interindividual and smartphone-induced variability in the data in the results section, and we have added a warning in the conclusions. Please also reply to comment 4a for further details.

## Reviewer’s comment 4a

*RESULTS:*

*The authors need to support a lot of inconsistencies seen in the findings reported:*

*a) Lines 198-199: Different configurations of phones with different microphone sensitivity, and models can bring in a lot of variability in data and questionability about the reliability of the data collected. Authors state that ‘36 % of the smartphones employed were made by Apple running iOS, the remaining 64% were from mixed brands running Android (33% Samsung, 16% Huawei, 10% Xiaomi)’ resounds the above concern.*

## Authors’ reply

In a citizen science experiment, data variability is expected to be substantially larger than in a controlled experiment performed in a lab by specialized personnel. The motivation for promoting such initiatives is clearly not accuracy. However our findings show that some general trends can be extracted from the data in an unambiguous way. In particular we have shown that applying calibration *ex post*, together with other precautions, can alleviate many issues. In particular we have shown that measuring quiet background and using sample calibration presets can be extremely helpful at extracting from the whole body of data subsets of more reliable records, providing therein self-assessment and self-correction features to the participatory experiment.

The numbers quoted by the reviewer correctly refer to the overall sample composition. However, when we consider calibrated measurements the ratio is very different: the majority of calibration tests were performed in our labs on iPhones, therefore the analysis of calibrated measurements was performed on phones running iOS in 72% of cases and Android in 28% of cases. We have reported these numbers in the revised manuscript, as they were not explicitly mentioned.

Stimulated by the reviewer’s comment we made an effort to quantify variance due to random effects (as opposed to variance due to the main effect, i.e. association between comfort and LAeq). We employed a generalized linear mixed model to estimate the variance due to the diversity in the smartphone models. We have added this analysis at the end of the results section.

## Reviewer’s comment 4b

*b) Lines 216 – 219: The authors state that : “In 70 cases (which contained valid data) the attribution to noisy and quiet states appeared to be inverted (i.e. both lower comfort rating and higher LAeq were attributed to the quiet state and vice versa). The labels for noisy and quiet states in these records were swapped”. This shows that the instructions were not correctly followed by the participants and this could be just one such instance. The data collected could be reflective of many such erroneous interpretations. The reasoning for inclusion and reversing the data, is not really satisfactory.*

## Authors’ reply

The reviewer's comment made us realize that we have not explained this point clearly. Incidentally we corrected a mistake as the “swapped” cases were in fact 65 instead of 70 (5 of these cases had already been dropped for other reasons).

As participants were asked to upload two sets of measurements, one for the "most comfortable" and one for the "least comfortable situation" the online submission form was partitioned into two distinct pages. In 65 cases participants appear to just have inverted the upload order. However they uploaded perfectly valid and consistent data. We could have labeled the pages neutrally as “observation 1” and “observation 2”. The observations can be classified univoquely from the reported comfort level alone. The “situation” layer allowed us to monitor how user-friendly the interface was. We have carefully checked that these mistakes did not affect the dataset. Their presence indicates that, for future use, the interface requires some simplifications. This case is just an example of a sanity-checking procedure which, we believe, is necessary to include in participatory science initiatives.

We have rewritten this point in the results section. We also added Table S2 showing that average comfort and LAeq for the database with 65 labels swapped and the one with the 65 cases completely removed do not change noticeably.

## Reviewer’s comment 5

*DISCUSSION: The authors discussion on the findings is elaborate and commendable. In my opinion although attempt has been made to categories the findings into different sub-headings, the readability is obscured due to the inclusion of results also into discussion. I suggest the authors rewrite this section with clear distinction of the results and only explain the findings in discussion, rather than putting in values and statistical analyses in this section. Two such instances are given below:*

*Lines 228-230: should be in results, not in included in discussion.*

*Line 462-463: Inferential statistics should be in results section*

## Authors’ reply

We thank the reviewer for his/her appreciation of our analysis. Following the reviewer's suggestion we have moved data presentation and statistical analysis from the discussion to the results section. These sections are marked in green in the working version of the revised manuscript. We hope the readability of the text has improved.
